# Qiviut cortisol in muskoxen as a potential tool for informing conservation strategies

**DOI:** 10.1093/conphys/cox052

**Published:** 2017-09-15

**Authors:** Juliette Di Francesco, Nora Navarro-Gonzalez, Katherine Wynne-Edwards, Stephanie Peacock, Lisa-Marie Leclerc, Matilde Tomaselli, Tracy Davison, Anja Carlsson, Susan Kutz

**Affiliations:** 1 Department of Ecosystem and Public Health, Faculty of Veterinary Medicine, University of Calgary, 3280 Hospital Drive NW, Calgary, Alberta, CanadaT2N 4Z6; 2 Department of Comparative Biology and Experimental Medicine, Faculty of Veterinary Medicine, University of Calgary, 3280 Hospital Drive NW, Calgary, Alberta, CanadaT2N 4Z6; 3 Department of Biological Sciences, Faculty of Science, University of Calgary, 507 Campus Drive NW, Calgary, Alberta, CanadaT2N 4V8; 4 Department of Environment, Government of Nunavut, P.O. Box 377, Kugluktuk, Nunavut, CanadaX0B 0E0; 5 Department of Environment and Natural Resources, Government of the Northwest Territories, P.O. Box 2749, Inuvik, Northwest Territories, CanadaX0E 0T0

**Keywords:** *Ovibos moschatus*, stress, hair, Arctic, liquid chromatography coupled to tandem mass spectrometry

## Abstract

Muskoxen (*Ovibos moschatus*) are increasingly subject to multiple new stressors associated with unprecedented climate change and increased anthropogenic activities across much of their range. Hair may provide a measurement of stress hormones (glucocorticoids) over periods of weeks to months. We developed a reliable method to quantify cortisol in the qiviut (wooly undercoat) of muskoxen using liquid chromatography coupled to tandem mass spectrometry. We then applied this technique to determine the natural variability in qiviut cortisol levels among 150 wild muskoxen, and to assess differences between sexes, seasons and years of collection. Qiviut samples were collected from the rump of adult muskoxen by subsistence and sport hunters in seven different locations in Nunavut and the Northwest Territories between 2013 and 2016. Results showed a high inter-individual variability in qiviut cortisol concentrations, with levels ranging from 3.5 to 48.9 pg/mg (median 11.7 pg/mg). Qiviut cortisol levels were significantly higher in males than females, and varied seasonally (summer levels were significantly lower than in fall and winter), and by year (levels significantly increased from 2013 to 2015). These differences may reflect distinct environmental conditions and the diverse stressors experienced, as well as physiological and/or behavioural characteristics. Quantification of qiviut cortisol may serve as a valuable tool for monitoring health and informing conservation and management efforts.

## Teaser

Muskoxen are increasingly exposed to multiple stressors that may impact their health and fitness. We measured stress hormones in their qiviut (wooly undercoat), and found differences across seasons, years and between sexes. Qiviut cortisol is a promising tool for guiding muskox conservation in a rapidly changing Arctic.

## Introduction

Climate change, taking place at an unprecedented pace in the Arctic, is resulting in multiple new stressors for wildlife (e.g. Kutz *et al.*, 2014), including increased heat stress (Ytrehus *et al.*, 2008, 2015; Van Beest and Milner, 2013), a higher frequency of extreme weather events (IPCC, 2013) and changes in exposure to pathogens (Burek *et al.*, 2008; Hoberg *et al.*, 2008; Ytrehus *et al.*, 2008, 2015; Kutz *et al.*, 2013, 2014). These stressors are having, and will continue to have, important impacts on wildlife (Altizer *et al.*, 2013; Post *et al.*, 2013; Ytrehus *et al.*, 2015). Muskoxen (*Ovibos moschatus*), large herbivores that reside at Arctic and subarctic latitudes, may be especially vulnerable to ongoing changes in the Arctic (MacCarthy *et al.*, 2001; Weller, 2005; Kutz *et al.*, 2017) as they are exceptionally well adapted to cold environments (Gunn and Adamczewski, 2003), but also have very low genetic diversity (Groves, 1997; MacPhee *et al.*, 2005).

Muskoxen are hunted for subsistence by aboriginal communities for whom they are a nutritious and affordable source of food, and serve as a key element in cultural traditions (Nuttall *et al.*, 2005). However, recent surveys in the Canadian North indicate that the two largest muskox populations, those on Banks and Victoria islands, Northwest Territories and Nunavut, have declined substantially, and, in some areas, are still declining (Nagy *et al.*, 2006, 2009a, 2009b; Davison *et al.*, 2013, 2017; Tomaselli *et al.*, 2016a). The cause of these declines remains uncertain, but is likely multifactorial, linked to icing events (Nagy and Gunn, 2009; Nagy *et al.*, 2009a), ecological changes associated with climate warming, and disease emergence (Kutz *et al.*, 2015, 2017; Tomaselli *et al.*, 2016b).

Ecological changes (e.g. climate change, habitat loss and fragmentation, fluctuations in food availability, human-caused disturbances, etc.) are increasingly recognized to be associated with chronic stress (chronic implying the stress occurs over long periods of time such as weeks to months), and may in turn lead to reduced health, fitness, and survival in free-ranging wildlife (Bonier *et al.*, 2009; Busch and Hayward, 2009; Ellis *et al.*, 2012; Koren *et al.*, 2012a). The stress response is mediated by the activation of the hypothalamic–pituitary–adrenal (HPA) axis, which leads to the secretion of glucocorticoids (GCs; mainly corticosterone or cortisol depending on the species) and subsequent mobilization of energy stores in mammals. While the short-term release of GCs plays an important role in allowing animals to cope with environmental change or challenges and to escape from life-threatening situations (Wingfield *et al.*, 1997; McEwen and Wingfield, 2003; Romero, 2004; Busch and Hayward, 2009), chronically elevated levels of GCs have been associated with physiological costs and detrimental effects including: increased susceptibility and vulnerability to diseases, a decline in immune responses, and reduced reproductive success (Wingfield *et al.*, 1997; Moore *et al.*, 2005; Acevedo-Whitehouse and Duffus, 2009; Busch and Hayward, 2009).

Hair, through its slow growth, is thought to give an integrated measure of GC concentrations over long periods of time, weeks to months, depending on the species-specific hair turnover rate (Sheriff *et al.*, 2011). Over the past decade, the measurement of GCs in hair and feathers has shown promise as a biomarker of long-term stress in a variety of wild species, in which hair and feather GC concentrations have been associated with important fitness and health characteristics as well as environmental factors. Hair GC levels, for example, have been negatively associated with proxies of fitness like body condition in polar bears (*Ursus maritimus*) (Macbeth *et al.*, 2012), and feather GC levels may be promising biomarkers of future survival in wild house sparrows (*Passer domesticus*) (Koren *et al.*, 2012a). Hair GC levels were also positively associated with certain long-term stressors like high hunting pressure in wolves (*Canis lupus*) (Bryan *et al.*, 2015), bile collection in Asiatic black bears (*Ursus thibetanus*) (Malcolm *et al.*, 2013), and low food availability in grizzly bears (*Ursus arctos*) (Bryan *et al.*, 2013). These studies suggest that hair GC levels may serve as a valuable tool to monitor wildlife health and inform conservation strategies.

The fur of muskoxen is one of their distinct features. They possess both a thick undercoat wool called qiviut, that is grown between early April and late November every year and shed in its entirety the following spring (between May and July), and long guard hairs that are produced continuously over several years and form the characteristic ‘skirt’ of muskoxen (Gray, 1987; Flood *et al.*, 1989; Mosbacher *et al.*, 2016). We propose that cortisol levels in qiviut may provide a measure of stress over the course of its growth, which in turn may give quantitative information about the health of the individuals and populations, and how they are affected by ecological changes in the Arctic.

In this study, we aimed to: (i) develop a reliable technique for cortisol quantification in the qiviut of muskoxen using liquid chromatography coupled with tandem mass spectrometry (LC–MS/MS), (ii) determine the natural variability in qiviut cortisol among wild muskoxen and (iii) assess the relationship between qiviut cortisol levels and sex, season and year of collection.

## Materials and methods

### Study area

**Figure 1:**
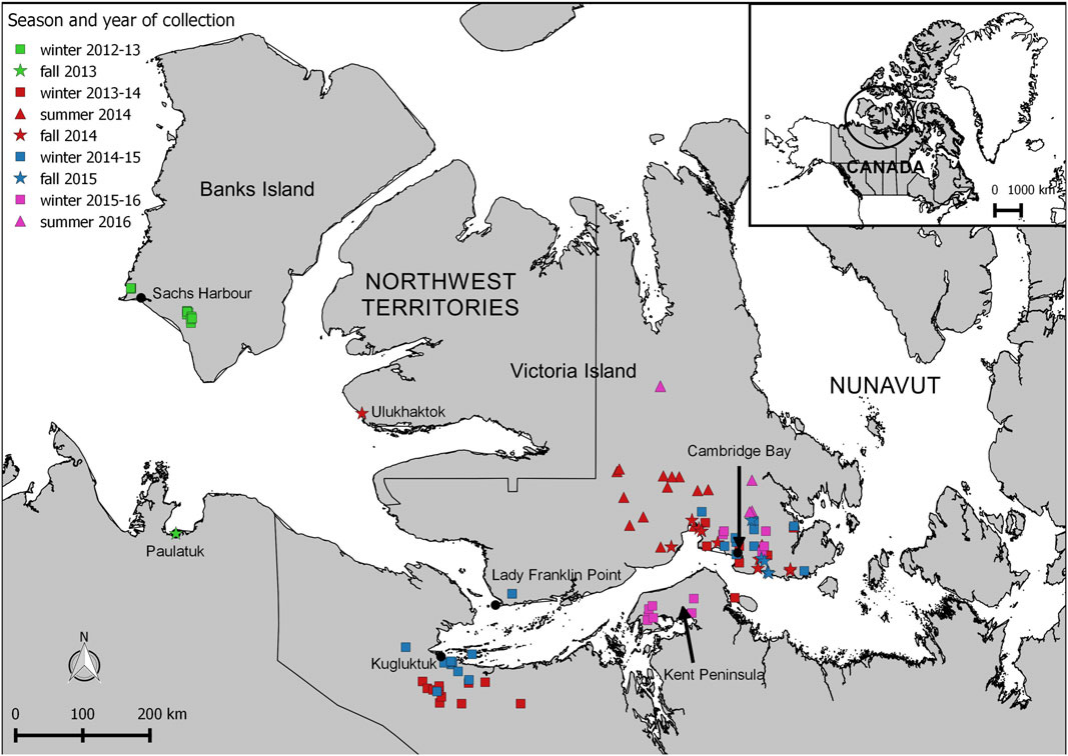
Map showing the location of the different communities from which muskox samples were obtained, and the geo-referenced hunting locations of the animals when available. Specific hunting location data were unavailable for muskoxen hunted in Ulukhaktok, Paulatuk, and for 17 of the 80 animals hunted in Cambridge Bay) (map generated in QGIS version 2.8.9).

Adult muskoxen harvested by subsistence and sport hunters were sampled from January 2013 to August 2016 in the Canadian Arctic near the communities of Paulatuk, Sachs Harbour and Ulukhaktok in the Northwest Territories (NWT), Kugluktuk and Cambridge Bay in Nunavut (NU), and away from communities on the Kent Peninsula and Lady Franklin Point (NU) (Figure 1 and Table 1). These locations were selected based on traditional muskox harvesting grounds and access to samples through collaborations with local Hunters and Trappers Committees (NWT) or Hunter and Trapper Organizations (NU), and sport hunt outfitters. Muskoxen harvested in Cambridge Bay and Lady Franklin Point belong to the Nunavut management unit MX-07, whereas those harvested in Kugluktuk and on Kent Peninsula are in the management unit MX-11. Each location was, however, treated separately, as they are geographically distant and characterised by different environmental conditions. Muskoxen from Lady Franklin Point and Kugluktuk are hunted by the community of Kugluktuk. Our collaboration with their Hunter and Trapper Organization gave us access to samples from community and subsistence hunts where both males and females are taken. Conversely, muskoxen from the Kent Peninsula and Cambridge Bay areas are hunted by the community of Cambridge Bay, with whom our collaboration gave us access mainly to samples from outfitted sport hunts where males are exclusively harvested. Muskox populations on Banks and Victoria Islands have declined substantially over the last decade, whereas those from the mainland sites have remained stable or increased (Davison and Williams, 2013; Davison *et al.*, 2013, 2017; Davison and Branigan, 2014; Leclerc, 2014; Tomaselli *et al.*, 2016a).

**Table 1:** Median and range of qiviut cortisol levels (pg/mg) in hunter-harvested muskoxen from Nunavut and the Northwest Territories represented by location, season and year of collection, and sex of the animal (*n* = sample size)

Location	Season and year of collection	Females median pg/mg (range)	Males median pg/mg (range)
Cambridge Bay	Winter 2013–14	-	11.3 (6.8–14.2)
			*n* = 6
	Summer 2014	-	8.2 (3.5–15.3)
			*n* = 15
	Fall 2014	18.8	13.61 (7.8–48.9)
		*n* = 1	*n* = 21
	Winter 2014–15	-	19.59 (13.9–30.3)
			*n* = 10
	Fall 2015	12.7 (6.1–18.5)	23.3 (3.6–27.1)
		*n* = 5	*n* = 9
	Winter 2015–16	-	21.4 (11.3–24.6)
			*n* = 8
	Summer 2016	-	9.24 (7.9–9.7)
			*n* = 4
Kent Peninsula	Winter 2014–15	-	10.5
			*n* = 1
	Winter 2015–16	-	15.3 (10.5–23.1)
			*n* = 8
Kugluktuk	Winter 2013–14	7.6 (4.3–38.3)	17.2 (6.9–22.5)
		*n* = 6	*n* = 6
	Winter 2014–15	12.2 (9.1–15.7)	21.8
		*n* = 8^a^	*n* = 1
Lady Franklin Point	Winter 2014–15	17.3	17.3 (13.7–20.9)
		*n* = 1	*n* = 2
Paulatuk	Fall 2013	7.73 (5.3–11.4)	5.51 (4.3–6.8)
		*n* = 7	*n* = 2
Sachs Harbour	Winter 2012–13	7.7 (4.2–14.5)	6.8 (12.5–20.2)
		*n* = 7^b^	*n* = 5
Ulukhaktok	Fall 2014	9.8 (7.4–15.5)	9.37 (5.6–14.0)
		*n* = 12	*n* = 5

^a^Six were pregnant.

^b^One was pregnant and three were lactating.

### Sample collection

Samples of muskox qiviut were obtained through individual subsistence and community hunts (Kugluktuk, Paulatuk and Sachs Harbour), individual subsistence and sport hunts (Cambridge Bay, Lady Franklin Point and Kent Peninsula), and the qiviut marketing pathway (Ulukhaktok). These are regular activities in the communities and no animals were culled specifically for our study. Samples were obtained under Animal Care and Use Permit #AC13-0121, the Wildlife Research Permit #2013-035, 2014-053, 2015-068 and 2016-058 for Nunavut, and the Wildlife Research Permit #WL500098, WL500158 and WL500257 for the Northwest Territories.

**Figure 2:**
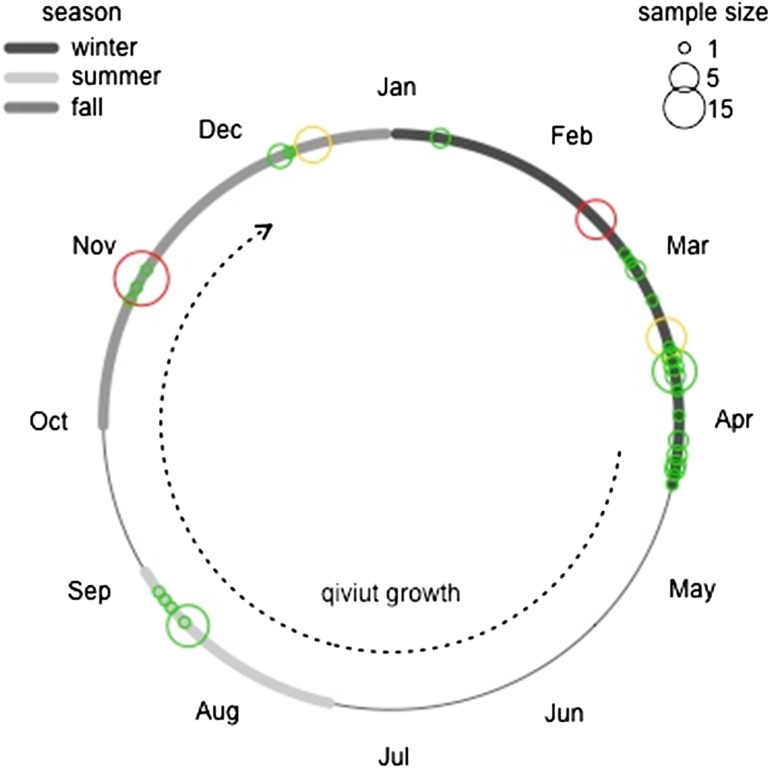
The annual cycle, showing the definition of winter, summer and fall seasons used in this study; the period of qiviut growth from early April to the end of November, and; the timing of qiviut collection for samples used in this study. The sample size (number of individuals) is indicated by the size of the circle, while the colour of the circle indicates the certainty in the date of sample collection (green: accurate to the day; yellow: accurate to the month; red: accurate to the season).

The timing of sample collection was directly linked to the traditional muskox harvesting seasons and samples were classified to different seasons (winter, summer or fall) based on their collection date. For the purposes of this study, samples collected between January and early April were considered winter samples, those collected late July through August were classified as summer, and those collected October through to mid-December as fall (Figure 2). Qiviut growth extends from early April to late November (Flood *et al.*, 1989). Thus samples tested in the fall would represent the end of the qiviut growth cycle, those from the summer the middle of the cycle, and the ‘winter’ samples would be a period of no qiviut growth (Figure 2).

### Gender determination

Hunters were asked to record the gender of the harvested muskoxen on a provided form. When this information was left blank or marked ‘unknown’, a skin sample was tested to determine the gender of the animal using laboratory genetic analyses that were performed within the Kyle Laboratory at Trent University, Peterborough, Ontario, Canada. DNA from each sample was extracted using the Qiagen DNAeasy Blood & Tissue Kit (Qiagen Inc., Mississauga, Ontario) following the recommended protocol supplied by the manufacturer. Gender was determined by amplification of the SRY and ZFX genes using the primers SRY-Y53-3C, SRY-Y53-3D (Fain and LeMay, 1995), ZFX-P2-3EZ and ZFX-P1-5EZ (Aasen and Medrano, 1990).

### Sampling procedures

Hunters were directed to sample the qiviut when skinning the animals by cutting a piece of hide measuring approximately 10 × 10 cm from the rump, near, but lateral to the base of the tail. The location of sample collection was standardized as hair cortisol levels may vary depending on the body region (Macbeth *et al.*, 2010; Ashley *et al.*, 2011; Terwissen *et al.*, 2013). Specifically, the rump was chosen because a sample can be taken without causing significant damage to the hide as it is along an edge that is cut during butchering. The sampling location is particularly important to consider when the hides are used by hunters for taxidermy or commercially for the fibre industry as it allows hunters to preserve the economic value. Additionally, guard hairs in this area grow continuously throughout the year (Flood *et al.*, 1989) and have been archived for future studies. Similar analytical methods can be used for guard hair cortisol quantification and guard hairs may also be used to assess the nutritional history of these muskoxen (Mosbacher *et al.*, 2016). Hunters and guides were expressly asked not to contaminate the fur with blood, urine or faeces, and samples that did not conform to the standardized sampling location or required quality were removed from this study (*n* = 4). For the samples from Ulukhaktok, hides were dried under ambient environmental winter conditions in the community, and then shipped to Calgary, Alberta, Canada from where they were exported for qiviut processing. In Calgary, hide samples were collected from the rump area, as per the hunter protocol. All samples were stored at −20°C until laboratory processing.

### Sample preparation

Qiviut samples from each animal were obtained by cutting guard hairs and qiviut away from the skin using a scalpel blade. Care was taken not to collect qiviut that was grossly contaminated with dirt, blood, urine or faeces and to not disturb the skin or collect hair roots. Guard hairs and qiviut were manually separated using clean forceps. Between 20 and 100 mg of qiviut were used for analysis, and each sample was run as a true experimental duplicate, collecting hair from the hide twice. Fifteen samples from Sachs Harbour were run in duplicate on two separate occasions to assess the repeatability of the method.

Muskoxen are cortisol-dominant in serum (Koren *et al.*, 2012c), and initial method development detected extremely low to non-quantifiable levels of corticosterone in qiviut, thus, only cortisol was quantified in this study.

Qiviut cortisol was quantified using LC–MS/MS. The use of LC–MS/MS eliminates potential issues of antibody cross-reactivity that are associated with enzyme linked immunosorbent assays (ELISAs) and has become the preferred method of hair steroid analysis in human clinical research (Handelsman and Wartofsky, 2013). It was previously applied by Koren *et al.* (2012a) to quantify testosterone, corticosterone and cortisol in the feathers of wild house sparrows, by Koren *et al.* (2012b) to quantify multiple steroids in serum samples from a wide range of captive wild mammals and birds (including muskoxen), and by Gesquiere *et al.* (2014) to measure testosterone concentrations in faecal samples from wild baboons (*Papio cynocephalus*).

The goal in sample preparation for LC–MS/MS is to provide as clean a steroid extraction as possible. For this reason, sample preparation differed from methods using ELISAs (Koren *et al.*, 2002; Davenport *et al.*, 2006; Macbeth *et al.*, 2010) in two ways: (i) the hair was not ground in a ball mill or cut with scissors and (ii) the sample preparation process was kept cold at all times (no stages in the washing and extraction were above 4°C). Both of these modifications were implemented to minimize mechanical disturbance and to maintain the surface oils, waxes and esters on the qiviut hair shaft in solid rather than liquid state, and with minimal surface area. As methanol is an excellent solvent for steroids, and the hair shaft is not thick, the methanol was expected to equilibrate and extract the steroid hormones present inside the hair shaft, while reducing potential matrix effects from other compounds on the hair. Thus, the concentrations of cortisol reported are the integrated sum of steroids external and internal to the hair shaft remaining after the cold washing procedure.

### Cold wash procedure to remove surface contamination

Each qiviut sample was placed in a 50 ml Falcon tube. A soap solution was prepared with 2 ml of Neutrogena Body Clear® Salicylic acid body cleanser for acne-prone skin (chosen for the specific claim that no residue was left after rinsing) added to 2 l of cold tap water and gently stirred for 3 min. The soap solution was then chilled on ice and 20 ml was added to each of the Falcon tubes containing the qiviut. The qiviut was washed by vortexing for 30 s, then the soapy water was decanted and the qiviut rinsed thoroughly with cold tap water, and patted dry in a clean paper towel. As a final rinse, the qiviut was next transferred to a new Falcon tube, and 20 ml of isopropyl alcohol (IPA) pre-chilled to −20°C, was added and mixed by gentle inversion for 10 s, then decanted to waste. Qiviut was air-dried in paper towel for at least 24 h before extraction.

### Extraction procedure

The qiviut samples were re-weighed before being placed in 13 × 100 mm borosilicate glass test tubes, and submerged under 6 ml of methanol pre-chilled to −20°C. A 100 μl spike of bio-identical, deuterated internal standard (IS—cortisol-d4 Catalogue D-5280, CDN isotopes, Pointe Claire, QC) in water/methanol (50/50, v/v) was added, with calibrators (Catalogue Q3880-000, Steraloids, Newport RI) and three quality control pools (low, medium and high calibrator pools in methanol) spiked at the same time. Samples were extracted for 20 h at 4°C in upright tubes (i.e. no vortexing or spinning). Cold supernatant was pipetted off the hair, transferred into new culture tubes, dried at 40°C under nitrogen (Techne® Sample Concentrator), and then stored, capped, at 4°C until reconstitution.

### Sample reconstitution

Each dry sample was reconstituted with 200 μl of water/methanol (100/100, v/v), and vortexed for 30 s. The total volume was transferred to a 600 μl microcentrifuge tube and centrifuged at 14 000 rpm for 20 min at 4°C. One hundred and fifty microlitres of supernatant were immediately transferred into LC autosampler vials for subsequent analysis.

Solid phase extraction (SPE) was not used as, during method development, we found a high correlation between the qiviut cortisol levels from twelve paired samples, whose analysis differed only by the addition or not of a SPE step (Pearson’s correlation coefficient *r*^2^ = 0.94, *P* < 0.001), and there was no significant difference between the mean qiviut cortisol levels obtained with or without SPE (paired samples *t*-test: *t* = −0.65, *P* = 0.53).

All samples were analysed by using an Agilent 1200 binary liquid chromatography (LC) system coupled to an AB SCIEX QTRAP® 5500 tandem mass spectrometer equipped with an atmospheric pressure chemical ionization (APCI) source in positive mode. LC separation was performed on an Agilent Poroshell 120 C18 column (50 × 3 mm, 2.7 μm particle size) at 45°C. The mobile phase A was water/methanol (75/25, v/v) and the mobile phase B was methanol/IPA (90/10, v/v). The 8.5 min gradient was 20–40% B (0–1.0 min), 40–60% B (1.0–5.0 min), 60–100% B (5.0–5.5 min), 100% B (5.5–6.5 min), 100–20% B (6.5–7.0 min) and held at 20% B (7.0–8.5 min). The flow rate was 0.6 ml/min and the injection volume was 20 μl. Nitrogen was used as the source, nebulizer and collision gas (curtain gas 28 psi; temperature 500°C; source gas 50 psi; collision gas medium; nebulizer current 8 μA). Mass resolution in Q1 and Q3 was set to unit resolution. Three transitions were monitored, the cortisol quantifier (–1), and qualifier (–2), and the deuterated cortisol IS quantifier transitions. Specific MRM (Multiple Reaction Monitoring) conditions were: *m*/*z* = mass-to-charge ratio (363.2/121.1; 363.2/115.1; 367.2/121.1), DP = declustering potential (all 85 V), EP = entrance potential (all 10 V), CE = collision energy (35; 114; 32 eV) and CXP = collision cell exit potential (all 20 V).

### Data processing

Cortisol peak integration was performed using Analyst 1.5.1 software (AB SCIEX). Sample quantitation used the area ratio between the analyte peak and the matched internal standard peak. Calibration curves (1/*x* weighted linear regression) covered the range from 0.25 through 250 ng/ml with *r*^2^ ≥ 0.99 in each run. The intra-assay variability was on average 8.3% and the inter-assay variability was 13.5%. Each cortisol result was divided by qiviut mass to obtain pg/mg. Duplicate quantitation was repeated on a second run, with new hair samples, whenever the coefficient of variation (CV), calculated as (standard deviation/mean) × 100 was ≥15%. The average from the two new replicates was then used for statistical analyses.

### Statistical analysis

Only adult animals, defined as two years or older based on hunter assessment, were included in the statistical analyses. To assess the effect of sex, season and year on log-transformed qiviut cortisol, we fit several different linear mixed-effects models with possible fixed effects including sex (male or female), year (2013, 2014, 2015 or 2016), season of collection (winter, summer or fall), and interactions between year and season, sex and season, and sex and year. In all models, we included a random effect for location of sampling to account for possible differences in qiviut cortisol among locations due to, for example, slight differences in hunter methodology or intrinsic differences in qiviut cortisol among locations linked to environmental (e.g. food quality, quantity and availability, weather etc.) or other location specific conditions. Qiviut cortisol levels were log-transformed to satisfy the linear model assumptions.

Models with different fixed effects were fit using restricted maximum likelihood and were compared using the adaptation of Akaike Information Criterion for small sample sizes (AICc) as the ratio of (sample size)/(number of parameters) was small (Burnham and Anderson, 2002). To avoid spurious relationships from fitting all possible combinations of fixed effects and interactions, models were developed in the following order: first, the effect of sex was tested, second the effects of year and season were assessed, and finally, the effect of sex was checked in the final model. This process resulted in testing a total of 14 different models (Table 2). The optimal subset of fixed effects that explained qiviut cortisol corresponded to the effects included in the model with the lowest AICc. This top model was re-fit using maximum likelihood to obtain the final parameter estimates. All models were fit using the R software (R Core Team, 2015) and the library lme4 for mixed-effects models (Bates, 2010). Marginal (fixed effects only) and conditional (fixed and random effects) coefficients of determination (*R*^2^) for mixed-effects models were calculated using the MuMIn library (Bartoń, 2015).

**Table 2:** Comparison of linear mixed-effect models including location as a random effect, with their corresponding AICc, ΔAICc in comparison to the best-fit model (bold), and degrees of freedom (DF)

Model fixed effects	AICc	ΔAICc	DF
**sex, year, season**	**158.86**	**0**	**9**
sex, year, season, sex:season	159.63	0.77	10
sex, year, season, sex:year	160.41	1.55	11
sex, season, year:season	162.50	3.64	12
sex, year:season	162.50	3.64	12
sex, year, year:season	162.50	3.64	12
sex, year, season, year:season	162.50	3.64	12
sex, year, season, sex:season, year:season	162.52	3.66	13
sex, year, season, sex:year, year:season	163.60	4.74	13
year, season	167.13	8.27	8
year, season, year:season	170.45	11.59	11
sex, season	171.94	13.08	6
sex, year	176.30	17.44	7
Sex	199.42	40.56	4

## Results

**Figure 3:**
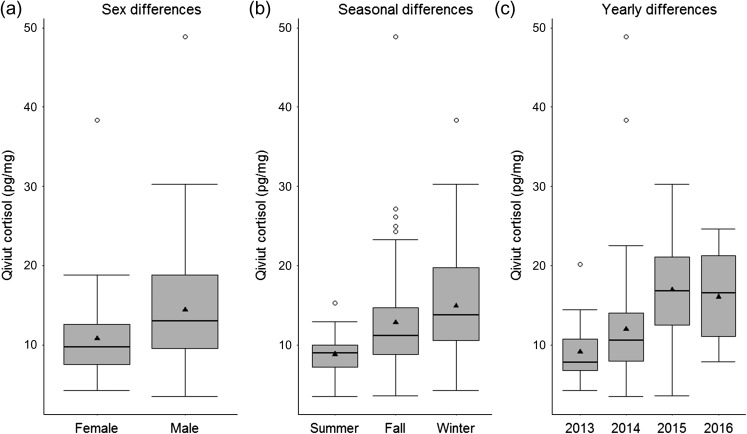
Boxplot showing combined qiviut cortisol values from all animals, seasons, years, sexes and locations by sex (**a**), season (**b**) and year (**c**). The thick horizontal lines correspond to the medians, the triangles to the means and the empty circles to the outliers.

Qiviut was sampled from 150 adult muskoxen between January 2013 and August 2016 (Table 1). Cortisol levels followed a right-skewed distribution and ranged from 3.5 to 48.9 pg/mg with a median of 11.7 pg/mg. This corresponds to an almost 14-fold variation. Qiviut cortisol levels by sex, season and year can be found in Fig. 3.

Model comparison using AICc showed similar support for three top models, all including the variables sex, year and season (Table 2). We based parameter estimates on the most parsimonious of these three, which had also the lowest AICc, and which included sex, year and season of collection as fixed effects, and no interaction terms (AICc = 158.86). The two other top models also included interactions between sex and season, and sex and year, respectively. Both the marginal and conditional *R*^2^ were relatively low at a rounded value of 0.33, respectively. The best-fit model conformed to the assumptions of normality and constant variance (see Supplementary data).

Based on the best-fit parameter estimates, qiviut cortisol levels were on average 1.30 (95% CI = 1.11–1.53) times higher in males than in females. Season also had a significant effect, with qiviut cortisol levels in fall and winter respectively 1.57 (95% CI = 1.26–1.95) and 1.70 (95% CI = 1.37–2.13) times higher on average than that in summer. Levels in fall and winter were not significantly different. Regarding differences between years, qiviut cortisol levels were on average 1.28 times (95% CI = 1.03–1.58), 1.67 (95% CI = 1.33–2.10) and 1.60 (95% CI = 1.21–2.10) times higher in 2014, 2015 and 2016, respectively, compared to 2013. Estimated qiviut cortisol values according to sex, season and year can be found in the Supplementary data.

The variance among locations was low (*σ*^2^_location_ = 0.0015) compared to the residual variation (*σ*^2^_resid_ = 0.16), but the conditional modes for the levels of the random effect at each location suggest slight differences in cortisol among locations that are consistent with the local muskox population trends, with qiviut cortisol concentrations that tend to be higher in declining populations on Banks and Victoria islands (Supplementary material).

## Discussion

### Findings

This is the first study measuring hair cortisol levels in muskoxen and it has provided important baseline data on variability within and among populations, sexes, seasons and years that will inform future studies.

Our first objective was to develop a reliable technique for cortisol quantification in the qiviut of muskoxen using LC–MS/MS. We chose to use LC–MS/MS as it has several advantages over the more commonly used ELISAs. It has a high specificity in molecular identification, as well as a calibration curve that is both linear and broad (in this case, 4 orders of magnitude). By contrast, in ELISAs, four-parameter logistic calibration curves will have quantitation errors outside of the linear portion of the curve. Therefore, when using LC–MS/MS, even when cortisol is present at a very low concentration, the detection of a signal corresponding to this hormone can be attributed to it with high confidence (Shackleton, 2010; Gale *et al.*, 2015). Additionally, although not reported in this study, multiple steroids can be quantified simultaneously with LC–MS/MS. This is particularly useful for wildlife studies as samples are often difficult to collect and thus gaining as much information as possible from a single sample is desirable (Shackleton, 2010; Koren *et al.*, 2012b). Furthermore, in LC–MS/MS, the addition of an internal standard to every sample (quality controls, unknowns and calibrants) early during their preparation process (just after the cold washing step in our case), intrinsically corrects for any subsequent analyte loss, and, therefore, improves the accuracy and precision of the method (Gale *et al.*, 2015). Finally, LC–MS/MS offers a better reproducibility in comparison with ELISAs that are poorly reproducible between laboratories (Shackleton, 2010). However, LC–MS/MS is expensive, and requires substantial technical expertise and highly specialized equipment. In contrast, ELISAs come with advantages, such as technical simplicity, considerable lower costs associated with the equipment, and easy transportation of the equipment to remote field locations (Crowther, 2009). The various advantages offered by each method render them complementary, with a valuable use in tandem. For example, LC–MS/MS could first be applied to prove the presence of a certain compound in a new sample type or species and to validate the ELISAs, which could subsequently be used more widely to expand the technique to a wider group of research teams that may have limited access to LC–MS/MS.

We found a high variability in qiviut cortisol levels among individual muskoxen, with levels ranging from 3.5 to 48.9 pg/mg, corresponding to an almost 14-fold variation. This wide range of values is consistent with other studies, where approximately 1.5 to 40-fold variations are reported depending on the study, number of animals included and species of interest (Table 3). This highlights the high inter-individual variability of hair cortisol concentrations within a species, which may reflect the different health and social status of individuals (Creel, 2001; Busch and Hayward, 2009; Dantzer *et al.*, 2014). Muskoxen are highly social and organized by a strong dominance hierarchy (Gray, 1987) and our results may, in part, reflect social interactions and status of individuals.

**Table 3:** Ranges of hair cortisol concentrations previously determined in free-ranging or captive wild mammal species using ELISAs (*n* = sample size)

Species	Median (Md) or mean x̅ pg/mg (range)	*n*	Reference
Rhesus macaque (*Macaca mulatta*)	x̅ = 110.3 (32.1–254.3)	20	Davenport *et al.* (2006)
Caribou (*Rangifer tarandus*)	Md = 2.31 (1.57–3.86)	12	Macbeth (2013)
Reindeer (*R. tarandus*)	Md = 2.88 (2.21–3.40)	12	Macbeth (2013)
Wolves (*Canis lupus*)			
Tundra-taiga	females: Md = 17.3 (9.95–32.2)	48	Bryan *et al.* (2015)
	males: Md = 15.8 (8.91–40.4)	55	
Northern boreal forest	females: Md = 14.6 (7.6–34.0)	24	Bryan *et al.* (2015)
	males: Md = 12.3 (4.8–26.8)	21	
Polar bears (*Ursus maritimus*)	x̅ = 9.5 (5.5–19.9)	17	Bechshøft *et al.* (2011)
	x̅ = 12.75 (3.98–24.42)	88	Bechshøft *et al.* (2012)
	Md = 0.48 (0.16–2.26)	185	Macbeth *et al.* (2012)
Grizzly bears (*Ursus arctos*)	Md = 2.84 (0.62–43.33)	151	Macbeth *et al.* (2010)
	Md = 8.1 (5.3–26.1)	113	Bryan *et al.* (2013)

Particularly high qiviut cortisol concentrations, greater than 28 pg/mg, were measured in four individuals. Although all samples were washed the same way, the potential remains for cryptic blood to persist on the hair surface and be extracted. Since blood concentrations of cortisol are considerably higher than hair concentrations, blood contamination could substantially increase measured results. However, these exceptionally high ‘outlier’ hides were re-tested and similar results attained, suggesting that these are real values, and not a result of undetected, local blood contamination. Moreover, these results are consistent with other studies where individuals with higher cortisol levels are commonly identified (e.g. Macbeth *et al.*, 2012; Malcolm *et al.*, 2013), and such outliers may represent highly stressed animals.

Although we were challenged by small sample sizes and a poor representation across groups (Table 1 and Supplementary data), we observed significant effects of year, season and sex on qiviut cortisol levels with a conditional *R*^2^ of 0.33. Other potentially relevant variables including, but not limited to, body condition, disease and pregnancy, or weather characteristics like snow depth, temperature and humidity may have contributed to the remaining 66% of the variability in qiviut cortisol levels but were not assessed in this study.

Qiviut cortisol levels varied by season, with levels in summer being significantly lower compared to fall or winter, and a non-significant increase from fall to winter. If we are measuring only a negligible part of the external cortisol, then based on the qiviut growth cycle (beginning in April and ending in November, with possibly slight latitudinal variation (Flood *et al.*, 1989)), August collected samples would reflect stressors from April to the time of collection and fall and winter collected samples would reflect the physiological stress experienced during the entire qiviut growth season. However, if external deposition of cortisol contributes substantially to the measured levels (see discussion on qiviut cortisol deposition in limitations and future considerations section below), we would predict that differences between fall and winter samples should reflect the season-specific stressors.

Seasonal variations in GC concentrations have been found in numerous ungulate species, often in association with environmental conditions (food abundance and quality, climatic characteristics, human activities, etc.). For example, faecal glucocorticoid metabolite (FGM) levels in bighorn sheep (*Ovis canadensis*) were significantly lower in the winter than in all the other seasons (Goldstein *et al.*, 2005). Similarly, among free-ranging elk (*Cervus canadensis*) in South Dakota, USA, FGM concentrations were at their lowest in winter and peaked in the summer, conceivably due to increased human disturbance, high temperatures, or normal seasonal metabolic patterns (Millspaugh *et al.*, 2001). Conversely, FGM levels peaked during the winter in a captive herd of red deer (*Cervus elaphus*) in Austria, with a significant effect of snow and minimum ambient temperature (Huber *et al.*, 2003). These contrasting results may be due to different responses among species to stressors or to variations in local environmental conditions. Faecal glucocorticoid metabolite levels were also significantly higher during the dry season in African elephants (*Loxodonta africana*) presumably due to less nutritious and more sparsely distributed sources of food, and an increased competition between individuals (Foley *et al.*, 2001). Similar seasonal patterns in FGM levels were described in zebras (*Equus quagga*) and springboks (*Antidorcas marsupialis*) during the dry season in relation to decreased food and water availability (Cizauskas *et al.*, 2015).

For muskoxen, stressors experienced between April and August may include heat extremes and insect harassment. Higher temperatures in the Arctic, particularly in the summer, have been described with increasing climate warming (MacCarthy *et al.*, 2001), and an outbreak of pneumonia in the muskox population of Dovrefjell, Norway during the summer of 2006 was associated with unusually high temperatures and humidity (Ytrehus *et al.*, 2008). Similarly, even though insect harassment effects on muskoxen have not been described, changes in insect phenology in the Arctic are already observed due to climate warming with an earlier occurrence, higher abundance, and an increased frequency of insect outbreaks, along with the observation of new species (Weller, 2005). Insect disturbance is a major stressor for caribou, and likely has negative effects on muskoxen as well. For example, oestrid fly and mosquito harassment affects the behaviour of caribou (*Rangifer tarandus*) and reindeer (*R. tarandus*) by significantly reducing their time spent foraging, and by augmenting their energy expenditure through increased movements and time spent standing (Toupin *et al.*, 1996; Vors and Boyce, 2009). It also has negative effects on reindeer body condition and productivity (Weladji *et al.*, 2003). Muskox females may experience additional stress associated with calving (mid-April to early June (Adamczewski and Flood, 1997; Gunn and Adamczewski, 2003)) and lactation. Female muskoxen lose much more fat during the first weeks of their lactation period following calving than during the previous 4 to 5 months (Adamczewski *et al.*, 1992). While this period is thought to be stressful for most ungulates, lactation was not associated with increased FGM levels in red deer (Huber *et al.*, 2003). Unfortunately, we were unable to sample female muskoxen during the summer, thus we cannot draw conclusions about this potential stressor from our data. Males may undergo additional stress due to the first agonistic interactions that are associated with the rut and begin in early to mid-July (Gray, 1987). Despite the multiple stressors occurring in summer, this season also corresponds to the period of highest food availability and quality in the Arctic, with a peak in July followed by a rapid decline (Gray, 1987; Adamczewski *et al.*, 1992), and would, therefore, be expected to be a season of low nutritional stress for muskoxen (Gunn and Adamczewski, 2003). This may contribute to explaining the lower qiviut cortisol levels observed in summer.

Through late summer and early fall, muskoxen experience social stress associated with breeding. Glucocorticoids peak during the breeding season in reptiles, amphibians, birds, and certain mammals (e.g. Touma and Palme, 2005). Reyes *et al.* (1997) reported a peak in serum cortisol concentrations in male pudu (*Pudu puda*) during the rutting season (Reyes *et al.*, 1997). Peak GC levels have also been reported during the breeding season for the northern muriqui (*Brachyteles arachnoids hypoxanthus*) (Strier *et al.*, 2003) and the tufted capuchin monkey (*Sapajus apella*) (Lynch *et al.*, 2002) and are attributed to the competition for breeding partners and an increased occurrence of aggressive encounters between males.

Beginning in the fall and extending into all of the winter period, muskoxen may experience stress due to higher human disturbance associated with hunting, low food quality and availability, and extreme cold temperatures. Anthropogenic stress may be higher than in the summer as people have more access to the land through the use of snowmobiles, thus increasing the disturbance of the animals (noise pollution and human encounters). Muskoxen react to snowmobile activity (McLaren and Green, 1985) and in the area around Cambridge Bay, behavioural changes, with animals having a much longer flight distance, have been observed with increased snowmobile activity (personal communication from Matilde Tomaselli, University of Calgary). Faecal glucocorticoid metabolite levels in elk (Creel *et al.*, 2002) and caribou (Freeman, 2008) are also positively associated with snowmobile activity. Lower food abundance and quality (Adamczewski *et al.*, 1992), along with increased agonistic behaviours (displacements) related to food accessibility often restricted to feeding craters (Gray, 1987), may lead to higher nutritional and social stress in the fall and winter. Muskoxen are highly adapted to the extremely cold environmental conditions in the Arctic, with one of the lowest metabolic rates recorded in ruminants both in the summer and winter, as well a high capacity to digest low-quality forage (Gunn and Adamczewski, 2003). However, reduced food intake and consumption of low-quality forage in the winter may be associated with increased cortisol concentrations indicating a shift towards a catabolic metabolism and the mobilization of energy stores (Parker and Rainey, 2004).

Qiviut cortisol levels were significantly higher in males than in females. However, there was also weak support for interactions between sex and season, and sex and year, so a more balanced sampling is required to confirm this finding. This difference contrasts with results from a previous study of hunter-harvested muskoxen which showed no significant difference in serum cortisol values between males and non-pregnant females aged more than one year (Wilcoxon rank sum test: *W* = 65, *P* = 0.98) (Katherine Wynne-Edwards, personal observation based on samples reported in Koren *et al.*, 2012c). However, as these were hunted muskoxen, any sex-specific differences may have been overshadowed by the immediate stress of hunting. Other studies have shown varied results regarding sex-specific differences in FGM levels. A study in captive goral (*Naemorhedus griseus*), one of the muskox’s closest taxonomic relatives, described higher FGM levels in males (Khonmee *et al.*, 2014), but many other studies in ungulates report no difference between sexes (captive red deer (Huber *et al.*, 2003), free-ranging elk (Millspaugh *et al.*, 2001), free-ranging American bison (*Bison bison*) (Ranglack *et al.*, 2016), and captive black (*Diceros bicornis*) and white (*Ceratotherium simum*) rhinoceros (Brown *et al.*, 2001)). Differences in FGM or hair GC levels between sexes may reflect distinct physiological and/or behavioural characteristics that certainly vary among species and across seasons.

We also observed a difference in qiviut cortisol levels among years. The trend for increasing cortisol from 2013 to 2015 suggests increasing population-level stressors. These may reflect environmental conditions (food availability, snow characteristics, temperature, humidity, occurrence of extreme weather events, predation risk, hunting pressure, etc.), exposure to pathogens, or a combination of these and other factors that could have affected both long and shorter-term stress levels. During this time period, the average annual surface air temperatures in Arctic Tundra region of Canada from 2013 to 2015 were 1°C, 1.1°C and 1.3°C above the 1961–1990 reference period, with average summer air temperatures 0.8°C, 1.1°C and 1.2°C, above the reference period (data from Environment Canada). These warm temperatures, and other associated ecological changes, may in part be contributing to increasing stress levels.

All the samples collected from 2013 to winter 2015 were analysed during the summer of 2015, whereas the samples collected onwards were analysed during the subsequent summer. Possible hormone degradation during storage may, therefore, have had a confounding effect on the yearly differences we observed. However, multiple studies, including a small pilot study by our team, have shown that cortisol is highly stable over long periods of time in hair at room temperature and in other matrices (e.g. serum, saliva and faeces) at subzero temperatures (Hunt and Wasser, 2003; Garde and Hansen, 2005; Stroud *et al.*, 2007; Macbeth *et al.*, 2012; Yamanashi *et al.*, 2016). These studies support that the differences in qiviut cortisol observed between years are real and not entirely driven by possible hormone degradation because of long-term storage conditions.

### Study limitations and future considerations

It is important to note that the hair cortisol levels we measured in muskoxen are not directly comparable to the levels measured in other species. Indeed, all the studies in Table 3 used one of two different ELISAs: the Oxford EA-65 Cortisol EIA kit, Oxford Biomedical, Lansing, MI, USA (Macbeth *et al.*, 2010, 2012; Macbeth, 2013) or the Salivary Cortisol ELISA Kit, Salimetrics, Philadelphia, PA, USA (Davenport *et al.*, 2006; Bechshøft *et al.*, 2011, 2012; Bryan *et al.*, 2013, 2015). A study comparing cortisol concentrations of both human and vervet monkey (*Chlorocebus pygerythrus*) hair measured using four different immunoassays (Alpco ELISA (Alpco, Salem, NH), DRG International ELISA (DRG Instruments GmbH, Marburg, Germany), Salimetrics ELISA (Salimetrics, LLC, State College, PA) and IBL luminescence immunoassay (LIA) (IBL International, Hamburg, Germany)) with ones measured using LC–MS/MS (Russell *et al.*, 2015), showed a high correlation (Pearson’s correlation coefficients were between 0.88 and 0.98, *P* < 0.0001), but hair cortisol concentrations were 2.5 to 20 times higher when measured using immunoassays than with LC–MS/MS. The Salimetrics ELISA and IBL LIA gave the closest results to LC–MS/MS with concentrations only approximately 2.5 times higher (Russell *et al.*, 2015). These results suggest that hair cortisol levels measured by LC–MS/MS and ELISAs are correlated, but not equivalent, and require validation against reference standards before comparison of concentrations across methods.

The qiviut cortisol concentrations we measured represent the sum of external and internal cortisol remaining after the cold washing procedure. The internal cortisol is assumed to be incorporated in the hair during the course of hair growth. It may derive both from circulating cortisol concentrations (Russell *et al.*, 2012) and from local synthesis in the skin (Ito *et al.*, 2005; Keckeis *et al.*, 2012). Because qiviut growth occurs from April to November, it is assumed that no additional cortisol enters the hair outside of this time period. However, cortisol originating from either blood, local production or both, and secreted by sebaceous and eccrine glands surrounding the hair follicle may be deposited on the outer cuticle of the hair shaft in association with sebum and sweat throughout the year (Raul *et al.*, 2004; Russell *et al.*, 2014). This external cortisol from sebum and sweat may, therefore, reflect more recent and ongoing events.

In this regard, however, qiviut might be less affected by cutaneous secretions than many other wildlife hair samples. In muskoxen, sebaceous glands and apocrine sweat glands are associated with the primary follicles that produce guard hairs, whereas no sweat glands and only small sebaceous lobules are associated with a minority of the secondary follicles that produce qiviut (Flood *et al.*, 1989). Qiviut is, consequently, a much dryer fibre than wool, with approximately 7% grease and a very low amount of suint (dried sweat), so the amount of cortisol from sebum and sweat on the hair shaft is probably limited (Rowell *et al.*, 2001; Helfferich, 2008). Nevertheless, our data, with a slight trend for increasing cortisol from fall to winter, suggests that recent events may be contributing to the cortisol levels measured.

Storage of qiviut prior to processing was not expected to affect the uptake of external cortisol. Qiviut samples were cut from pieces of hide that were stored at −20°C and taken out only for the duration of sample cutting. Condensation and humidity due to thawing may have increased the permeability of the hair, as water is known to extend the hair cuticle, potentially causing the cortisol from sebum, sweat and possible external contaminants (blood, urine, faeces) to leach inside the hair shaft, thereby confounding our results (Kidwell and Blank, 1996; Macbeth, 2013; Cattet *et al.*, 2014). Once this external cortisol has been incorporated into the hair matrix, it cannot be removed by decontamination protocols (Kidwell and Blank, 1996). Its contribution to the concentrations measured in our study remains unknown but is likely consistent across study areas and time periods as samples all experienced an initial freezing event.

Additional research is needed to provide evidence that qiviut cortisol levels reflect adrenal activity, which will enable us to link high qiviut cortisol levels with stress. ACTH challenges involving repeated weekly injections over several weeks have been used to validate that the measurement of cortisol in hair reflects adrenal changes in several species of domestic or captive wild animals, including eastern chipmunks (*Tamias striatus*) (Mastromonaco *et al.*, 2014), Canada lynx (*Lynx canadensis*) (Terwissen *et al.*, 2013) and domestic cattle (*Bos taurus*) (González-de-la-Vara *et al.*, 2011). By contrast, a study in caribou and reindeer showed that hair cortisol levels were not affected by a single ACTH injection (Ashley *et al.*, 2011). Consequently, in order for changes in adrenal activity to be detectable in the hair, it seems necessary to administer a prolonged ACTH challenge (e.g. including repeated injections over time), mimicking chronic stress. Such a validation study is a focus of our future work.

Finally, the determination of the qiviut growth cycle was based on the observation of captive animals in Saskatoon, Saskatchewan, Canada. The exact timing of the qiviut growth cycle in wild muskoxen in the Arctic remains unknown, but is unlikely to differ substantially as qiviut is shed at a similar time (Flood *et al.*, 1989; Gray, 1987). Qiviut growth rate varied throughout the cycle in captive muskoxen in Alaska, USA, with a peak in August and a slow-down in October, although growth was observed until the end of November (Robertson, 2000). The differences in qiviut growth rate throughout the cycle or among individuals may affect cortisol deposition in the qiviut hair shaft. A better understanding of the qiviut growth patterns is important for interpretation of results and seasonal data.

## Conclusion

The Arctic ecosystem is currently experiencing one of the greatest rates of climate and ecological change in the world (Overland *et al.*, 2016). How Arctic-adapted species will persist in the face of this rapidly changing environment and increasing cumulative stressors is of concern, both from a conservation and human point of view, as these species have important economic, nutritious and socio-cultural values for Arctic communities (Nuttall *et al.*, 2005; Kutz *et al.*, 2014). The health of many Arctic mammal species is already changing, and is predicted to be increasingly affected by climate warming and its multiple impacts (loss of sea ice, increased occurrence of extreme weather events, elevation of sea level, higher air temperatures, etc.), which may alter food webs and pathogen transmission patterns (Weller, 2005; Burek *et al.*, 2008; Altizer *et al.*, 2013; Post *et al.*, 2013; Kutz *et al.*, 2014). There is a crucial need, therefore, to develop robust methods for monitoring individual and population health of wildlife species in order to inform management. Research and surveillance of wildlife species is challenging, with many logistic and financial restrictions, along with an access to often restricted sample sizes and diagnostic tools (Ryser-Degiorgis, 2013). These difficulties are exacerbated in remote locations like the Arctic, where the environment is extreme (Curry *et al.*, 2011). Hair sampling could be incorporated with minimal additional effort into community-based wildlife health surveillance programmes, notably through the collaboration with hunters for sample collection, and without major financial constraints (Brook *et al.*, 2009; Carlsson *et al.*, 2016). We have shown that qiviut GC levels can be reliably quantified in muskoxen using LC–MS/MS, and found a high inter-individual variability in qiviut cortisol levels, along with sex, seasonal and year effects. These results suggest that qiviut cortisol may become a valuable tool for monitoring individual and population health in muskoxen and informing conservation and management efforts.

## Supplementary Material

Supplementary Data
