# Supplementary material for: Qiviut cortisol in muskoxen as a potential tool for informing conservation strategies
Source: Conserv Physiol. 2017 Sep 15;5(1):cox052. doi: 10.1093/conphys/cox052 (PMC5601961; doi:10.1093/conphys/cox052)

**Supplementary data:**

**Table 1**: Parameter estimates of the final model explaining log-transformed cortisol levels and including sex (male versus female), year (2014, 2015, and 2016 versus 2013), and season (summer and winter versus fall) as fixed explanatory variables.

|  | **Parameter estimate** | **Standard Error** | **t value** |
| --- | --- | --- | --- |
| **(Intercept)** | 1.5453 | 0.1436 | 10.7585 |
| **sex-male** | 0.2656 | 0.0800 | 3.3194 |
| **year-2014** | 0.2435 | 0.1106 | 2.2011 |
| **year-2015** | 0.5142 | 0.1163 | 4.4218 |
| **year-2015** | 0.4682 | 0.1399 | 3.3467 |
| **season-fall** | 0.4513 | 0.1104 | 4.0886 |
| **season-winter** | 0.5334 | 0.1129 | 4.7257 |

**Table 2**: Estimated mean (95% CI) qiviut cortisol levels per sex, season and year*.

| **Year** | **Season** | **Sex** | **Mean (95% CI)** |
| --- | --- | --- | --- |
| 2013 | winter | male | 10.43 (8.36-13.01) |
|  |  | female | 7.99 (6.41-9.97) |
| 2014 | winter | male | 11.65 (9.33-14.53) |
|  |  | female | 8.93 (7.16-11.14) |
|  | summer | male | 6.83 (5.50-8.49) |
|  | fall | male | 10.73 (8.64-13.32) |
|  |  | female | 8.23 (6.63-10.21) |
| 2015 | winter | male | 11.71 (9.39-14.61) |
|  |  | female | 8.98 (7.20-11.20) |
|  | fall | male | 10.79 (8.69-13.39) |
|  |  | female | 8.27 (6.66-10.27) |
| 2016 | winter | male | 11.99 (9.61-14.96) |
|  | summer | male | 7.03 (5.35-9.25) |

* Only those combinations of factors for which we had data (Table 1) are shown.

**Table 3**: Sample size description per season, year and sex.

*Samples collected in a single location (other than Cambridge Bay); †Samples collected only in Cambridge Bay

|  |  | Female | Male |
| --- | --- | --- | --- |
| Winter | 2013* | 7 | 5 |
|  | 2014 | 13 | 6 |
|  | 2015 | 13 | 9 |
|  | 2016 | 0 | 16 |
| Summer | 2013 | 0 | 0 |
|  | 2014† | 0 | 15 |
|  | 2015 | 0 | 0 |
|  | 2016† | 0 | 4 |
| Fall | 2013* | 7 | 2 |
|  | 2014 | 13 | 26 |
|  | 2015† | 5 | 9 |

**Figure 1:** Plot showing the residuals against the fitted values.


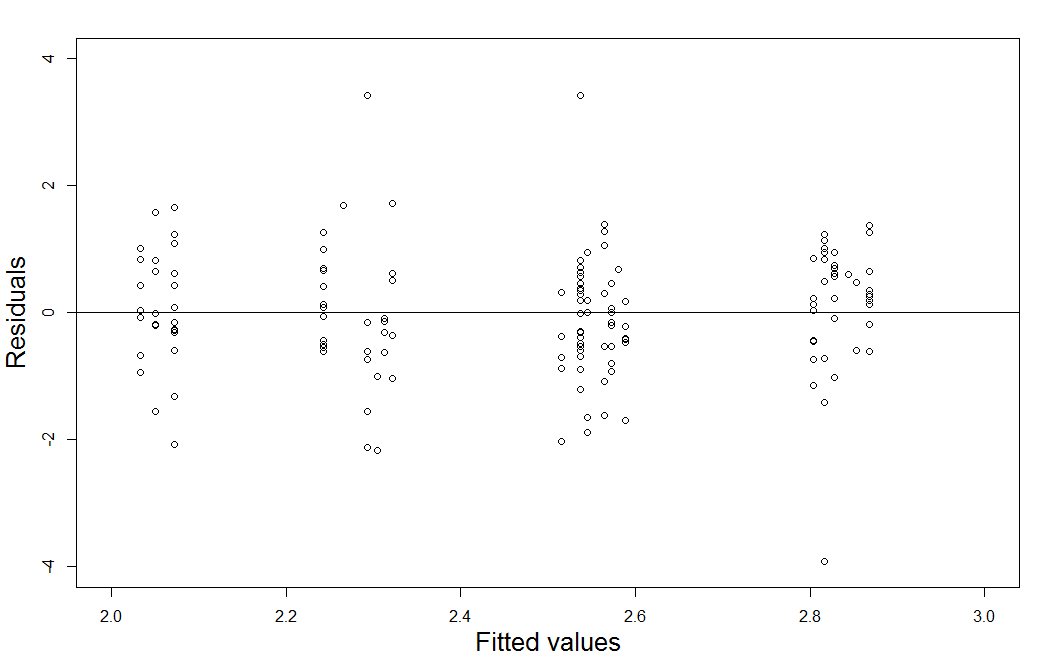


**Figure 2:** Plots showing (a) the conditional modes for the random effect level (i.e., estimated mean difference from the mean, conditional on the fixed-effects) for each location of sampling and (b) the conditional modes and the residuals on the same scale.


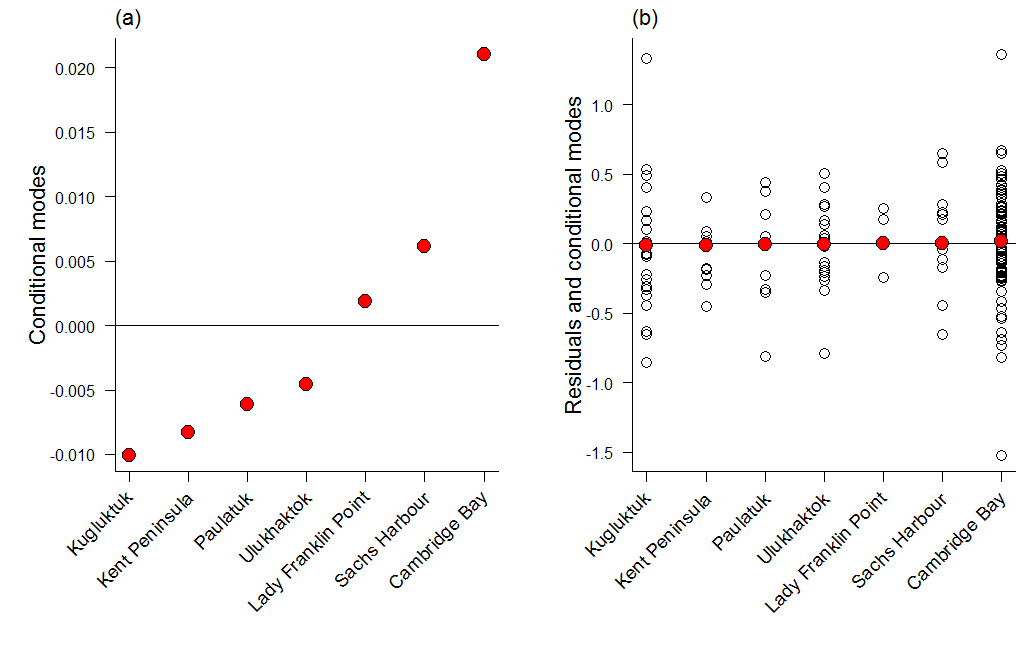

Supplement: Supplementary Data [file SupplementaryDataReviewed.doc]
